# The Fate of Patients Who Started Hemodialysis during Childhood or Adolescence: Results of an Interregional Moroccan Survey

**DOI:** 10.1155/2014/389729

**Published:** 2014-12-10

**Authors:** F. Z. Souilmi, T. Sqalli Houssaini, G. EL Bardai, N. Kabbali, M. Arrayhani, M. Hida

**Affiliations:** ^1^Pediatric Department, Hassan II University Hospital, Sidi Harazem Road, 30000 Fez, Morocco; ^2^Faculty of Medicine and Pharmacy, Sidi Mohamed Ben Abdellah University, Sidi Harazem Road, 30000 Fez, Morocco; ^3^Nephrology Department, Hassan II University Hospital, Sidi Harazem Road, 30000 Fez, Morocco

## Abstract

Hemodialysis is the most used renal replacement therapy for children in Morocco. The objective of this study was to determine the prevalence of patients who started hemodialysis in childhood and study their characteristics and specificities of their care. For this we conducted a multicentric descriptive cross-sectional study of all chronic hemodialysis patients who started dialysis in pediatric age, in hemodialysis centers in four of the sixteen regions of Morocco. We collected 2066 patients undergoing dialysis in 39 hemodialysis centers; from these, only 72 patients (3.48%) started hemodialysis in childhood. The average age of patients was 20.64 ± 6.5 years with a sex ratio of 1.9. Duration of dialysis was 78.2 ± 56 months. The cause of end stage renal disease was urological abnormalities in 18% of cases and glomerulopathy in 12.5% of cases; however, it remains unknown in half of the patients. Over 18 years, 74% of patients are without profession, it is active in 13% of cases, and pursuing studies are only in 13% of cases. Patients under 5 years and those with a low weight are rarely taken care of in chronic hemodialysis with little individualization of prescription. Greater attention should be paid to renal transplantation that is desired by the majority of these patients (92%).

## 1. Introduction

Hemodialysis (HD) is the most used renal replacement therapy for children in Morocco. If the adult population is the majority of patients followed, many hemodialysis centers host or had hosted children whose care differs from that of adults. The aim of this study was to determine the prevalence of patients who started hemodialysis in childhood and study their characteristics and specificities of their care.

## 2. Methods

This is a multicentric descriptive cross-sectional study achieved during the first quarter of 2012. We studied all patients undergoing a chronic hemodialysis (CHD), who have started dialysis in childhood (before age of 18) and are still hemodialyzed in public or private hemodialysis centers. Our survey included all centers in the following regions: Meknes-Tafilalet, Fez-Boulemane, Taza-Al Hoceima-Taounate, and the Oriental regions. These four regions are among the sixteen regions of Morocco. A questionnaire was given to patients and to the nephrologists working in these centers by physician investigators belonging to the University Hospital Hassan II of Fez. These investigators moved to the different centers so as to collect demographic, clinical, and paraclinical data concerning all the patients included in study. The given questionnaire also helped us to collect their opinions on renal transplantation and the reasons for the position they have taken. Then, the data collected was processed by the statistical software (SPSS statistics version 17.0) under the license of Faculty of Medicine and Pharmacy of Fez. Qualitative variables were expressed as percentages and quantitative variables as mean with standard deviation.

## 3. Results

**Figure 1:**
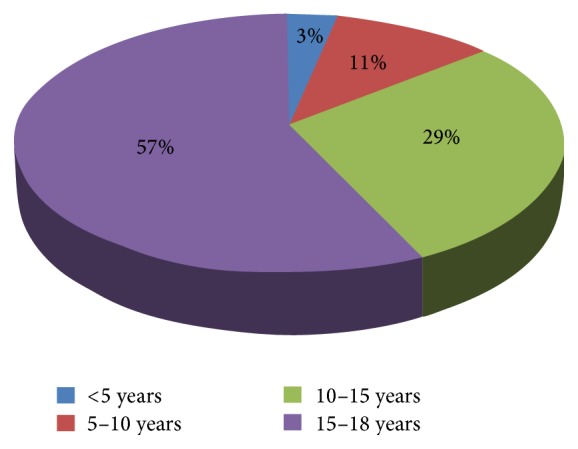
Distribution of patients according to the age of the beginning of hemodialysis.

A total of 2066 patients undergoing dialysis in 39 hemodialysis centers (22 public and 17 private centers) were included. From these patients, we collected only 72 patients (3.48%) who started hemodialysis in childhood: 38 patients in private centers and 34 patients in public centers. We noted a male predominance with a sex ratio of 1.9 (47 M/25 F). The mean age of our patients was 20.6 ± 6.5 years (4–35 years), while the average age concerning the beginning of dialysis was 14.12 ± 3.4 years, with an age extreme ranging from 3 to 18 years (Figure 1). The mean hemodialysis duration was 78.2 ± 56 months (Table 1). The initial nephropathy is urological abnormalities in 18% of cases, a glomerulopathy in 12.5% of cases, and a systemic disease in 7% of cases. Hereditary nephropathy was diagnosed in 4 patients (5.5%) and hypertensive nephropathy in 2 patients only (3%). However, the etiology was unknown in 54.2% of cases (Table 2). The number of dialysis sessions per week was three sessions in 49 patients (68%) and two sessions in 23 patients (32%). All the patients are hemodialyzed via a native arteriovenous fistula (AVF) except one child who had a permanent hemodialysis catheter. In 69.40% of cases, it is their first arteriovenous fistula (AVF). It is done at least twice in 30.6% of cases. The percentage of patients having 3 sessions per week has increased from 40% for patients whose age is less than 10 years (this age is usually characterized by a low weight: less than 30 kg) to 65% in the 10 to 15 age group and 73% in the age group of patients who are over 15 years old (Table 3). The female patients, who have reached adulthood, suffer from growth retardation in 43.50% of cases with a size ≤ 150 cm (=−2SD) and a BMI = −2SD in 8.70% of cases, whereas the male patients, who have reached adulthood, suffer from a growth retardation in 60% of cases with a size ≤ 160 cm (=−2SD) and a BMI = −2SD in 23.2% of cases.

Among 75% of our patients who have reached the age of 18 years, 2% have a primary level of study and 35% reached a college level, 39% a secondary school, and 17% a high school while 7% are illiterate. Patients are unemployed in 74% of cases and working in 13% of cases. Two of the 49 patients, who have reached adulthood, are married. Concerning the patients on hemodialysis whose age is under 18 (25%), 22% of them study at a secondary school, 6% of them study in a high school, and 72% are out of school. No patient received a renal transplantation. Patients would like to be transplanted in 92% of cases. Their main motivation factors were related primarily to fluid restriction and diet constraints (52%), studies or work difficulties (62%), marital problems and desire to have children (30.50%), complications of hemodialysis (21%), free time (17%), and travel (10%). Three reasons are associated in 6% of cases and four reasons are associated in 25% of cases. 8.33% of patients refused renal transplantation mainly because of the presumed high cost of the operation. The patients were informed about renal transplantation by their physician in 48% of cases, close contacts in 17% of cases, and the media in 15.5% of cases. Once the subject was explained in this investigation, 80.5% of patients agreed kidney transplantation from a living donor, among which 48.2% claim to have a potential related living donor. In 20.68% of cases, kidney transplantation from a cadaveric donor was denied.

## 4. Discussion

In the case of children and adolescents, the registers data of the chronic kidney disease (CKD) are limited since there are low reference populations. This means that it is difficult to have direct comparisons of rates of CKD of childhood (incidence and prevalence) in different geographical areas of the world because of many reasons among which we find the methodological differences in the age groups of the studies, the characterization of the degree of renal failure, and the classification of disease [1]. The only studies available are those dealing with the prevalence and incidence of CKD in relation to the population of healthy children, which make the comparison with some of our data not applicable. In Morocco, according to the census of the population in 2004 published by the High Commission for Planning, the total population of the four regions covered by the study represents 29.50% of the Moroccan population. The pediatric population under 19 years old represents 41.80% in these regions and 24.95% of the pediatric population in Morocco [2].

Public health services and social benefits available in these 4 regions are similar to the remaining 12 regions in Morocco. In addition, at the time of the study, four university hospitals were functional, respectively, in the cities of Rabat, Casablanca, Marrakech, and Fez. Each center serves 4 regions of Morocco and a comparable number of patients. However, the provision of care in the private sector is larger in some other parts of the country, particularly in the cities of Casablanca (the largest city in Morocco) and Rabat (the capital).

The Magredial register data (Register of the End Stage Renal Disease: Morocco-Transplant-Dialysis) involving four regions (those involved in our study are not included) shows that, in 3112 patients undergoing renal replacement therapy, the age group ranging from 0 to 19 years represents 6.50% of cases [3]. Few data are available in the North African countries, in the Middle East, or in Central African countries since there are no regional registers which could help the authorities to collect and publish valid epidemiological data. The only data (incidence and prevalence) that come from these developing countries are mainly derived from reports of tertiary centers of care [1, 4]. In different registers, the incidence of ESRD varies from 7.2 to 16 new cases per year and per million of the age-related population (PMARP) according to the age limit (15 to 20 years old) [4]. According to NAPRTCS register (North American Pediatric Renal Trials and Collaborative Studies), ESRD mainly affects children between the ages of 6 and 18 years, with the highest incidence in children whose age is between 15 and 19 years old (28 PMARP) [5, 6]. However, in our study the largest number of cases was between 10 and 18 years old.

In most studies, the prevalence and the incidence of the predialysis stage of CKD or replacement therapy are higher among boys than girls [1]. In Morocco, the sex ratio is 1.25 for children under 19 years old [3]. These results can be explained by the high incidence of congenital anomalies of the kidney and urinary tract among boys (CAKUT) [1, 5, 7]. The average age of our patients as they started dialysis was 14.12 ± 3.4 years and only 3% of them were younger than 5 years. The reason behind which we have few children aged less than 5 years old in this study is that they are generally not considered as eligible for dialysis or kidney transplant. From the age of 15 years, children are considered as adults and are then treated more easily. Therefore, these data are similar to the initial experience of some countries [8]. Arteriovenous fistula (AVF) has often been done twice at least, probably due to the nonsystematic use of microscope. Microsurgery is necessary for the creation of a functional AVF for most children, but few surgeons are trained for this technique, which is rarely used even in other countries [9]. Conventional hemodialysis is usually performed three times per week with a duration which cannot be less than four hours per session [10]. Because of the lack of financial resources or dialysis machine available in dialysis centers, 32% of our patients received only two sessions per week or 8 hours of dialysis per week. In this survey, urological abnormalities (18%) are the most common causes of the ESRD. The urological abnormalities represents also the most found etiology in our country (34% of cases) as well as in different countries (USA 57% of cases, Italy 68% of cases, India 47% of cases, and Iran 52% of cases) [7, 11–13]. Glomerular diseases (12.5% in this series) represent the second etiology of ESRD, with a very variable rate depending on the country. These diseases are found in Europe and the United States in percentages ranging from 7% to 24.5% of cases. In other countries such as Vietnam, Nigeria, and South Africa, glomerulonephritis represents the main cause of ESRD with 66.4%, 58.3%, and 56.4%, respectively. This difference is due to a higher prevalence of infectious diseases [1, 3, 5, 7, 11]. Hereditary nephropathies (5.5%) represent a small percentage in comparison with other studies that found a higher incidence (we have nationally 17% of cases and Iran has 21.7%, Tunisia 29%, Italy 25%, Iraq 21.3%, and Jordan 29.7%) in relation with the frequency of intermarriages in these countries [4, 7, 8, 12]. The etiologies we have found may be underestimated because of the very high percentage of unknown etiologies (54%) in this study in comparison with other studies. This testifies that the management of the patients' care is done in late and advanced stages of their kidney disease [5–8, 11]. Growth retardation is noticed in children with CKD. The establishment of a treatment with the recombinant human growth hormone (rhGH) improves growth rate of these children; however, the recognition and the early treatment of malnutrition, mineral and bone disorders, and electrolyte and acid-base abnormalities should take place before considering a growth hormone (GH) treatment [10, 14, 15]. In our study, no patient was treated with growth hormone which reflects an inadequate management of hemodialyzed children in adult hemodialysis centers. The social integration and the quality of life of our patients are poor as it is shown through their low level of education, their social status (2/49 are married), and their professional status (74% have no profession or work). The young adults who had a prolonged dialysis in childhood are more likely to be unemployed than the age-matched population and those who are employed have a lower professional qualification [14, 16]. Many factors contribute to this observation: frequency of hemodialysis sessions, complications of kidney disease and dialysis, and higher incidence of developmental disorders in comparison with the general pediatric population, with an impact on neurodevelopment and psychosocial outcome [17]. In the long term, the risk of mortality of children undergoing dialysis is thirty times higher than normal children [14, 18]. In the pediatric population, different studies emphasize on the fact that the younger the age of the patient at the beginning of dialysis is, the higher the mortality is, characterized by a higher incidence of comorbidities. However, mortality is lower in children than adults who are undergoing dialysis [14, 19, 20]. Nevertheless, Mortality is much higher (4 to 10 times) in children undergoing dialysis than those with kidney transplantation. Indeed, the expected remaining life for dialysis children, aged from 0 to 14 years, is only 18.3 years, against 50 years for kidney transplantation children [1, 14]. Furthermore, renal transplantation provides a better chance of rehabilitation in terms of training and psychosocial functioning [9]. This highly desired therapy by our hemodialyzed patients remains difficult to access despite the expended efforts to develop the field of transplantation in our country.

A national kidney transplant program was launched in Morocco in 1998. Since then, about 360 kidney transplants have been performed with more than 90% from related living donors. The only transplant center in the four regions of our study is that of the University Hospital of Fez. Sixteen renal transplants have been performed in this center. One of them was conducted in 2014 in an adolescent under the age of 13 years. A total of about 20 pediatric kidney transplants have been performed to date in Morocco, mostly in adolescents. A recent program of the Department of Health is looking to expanding the supply of transplantation in Morocco and provides a pediatric priority.

## 5. Conclusion

This study has allowed us to achieve initial assessment and observation on the management of pediatric hemodialysis in Morocco. It reveals that the hemodialysis centers, which are mainly intended for adults, do not take into consideration the pediatric specificities of hemodialyzed children. The development of pediatric renal transplantation will surely contribute to the improvement of the prognosis and the quality of life of patients who had started hemodialysis in childhood.

## Figures and Tables

**Table 1:** Mean duration in hemodialysis according to the age when it began.

The age at the beginning of HD	Mean duration of HD (months)
<10 years	29,5
10–15 years	93,1
15–18 years	80,1

**Table 2:** Etiologies of ESRD.

Initial nephropathy	Number of patients (%)
Unknown nephropathy	39 (54%)
Chronic tubulointerstitial nephritis/uropathy	13 (18%)
Glomerulonephritis	9 (12,5%)
Systemic disease	5 (7%)
Hereditary nephropathy	4 (5,5%)
Hypertensive nephropathy	2 (3%)

**Table 3:** Number of sessions of hemodialysis per week depending on the age.

	2 sessions	3 sessions
≤10 years	6 patients	4 patients
10–15 years	10 patients	19 patients
>15 years	9 patients	24 patients

## Abbreviations

AVF: Arteriovenous fistula
BMI: Body mass index
CKD: Chronic kidney disease
ESRD: End stage renal disease
F: Female
HD: Hemodialysis
M: Male
PMARP: Per million of the age-related population
rhGH: Recombinant human growth hormone.
